# Improvement of Peptide Affinity and Stability by Complexing to Cyclodextrin-Grafted Ammonium Chitosan

**DOI:** 10.3390/polym12020474

**Published:** 2020-02-19

**Authors:** Andrea Cesari, Alessandra Recchimurzo, Angela Fabiano, Federica Balzano, Nicolò Rossi, Chiara Migone, Gloria Uccello-Barretta, Ylenia Zambito, Anna Maria Piras

**Affiliations:** 1 Department of Chemistry and Industrial Chemistry, University of Pisa, via G. Moruzzi 13, 56126 Pisa, Italy; andrea.cesari@outlook.com (A.C.); alessandra.recchimurzo@phd.unipi.it (A.R.); gloria.uccello.barretta@unipi.it (G.U.-B.); 2 Department of Pharmacy, University of Pisa, via Bonanno Pisano 6, 56126 Pisa, Italy; angela.fabiano@unipi.it (A.F.); rossiniko94@hotmail.it (N.R.); chiaramigone@gmail.com (C.M.); ylenia.zambito@unipi.it (Y.Z.)

**Keywords:** chitosan, cyclodextrin, macromolecular complex, dalargin, enkephalin, chymotrypsin, NMR, oral peptide delivery, peptide stability

## Abstract

Cyclodextrin-grafted polymers are attractive biomaterials that could bring together the host–guest complexing capability of pristine cyclodextrin and the pharmaceutical features of the polymeric backbone. The present paper is aimed at characterizing the potential application of ammonium–chitosan grafted with 2-methyl-β-cyclodextrin (N^+^-rCh-MCD) as the functional macromolecular complexing agent for the oral administration of the neuropeptide dalargin (DAL). Specific NMR characterization procedures, along with UV and fluorescence techniques, as well as biological in vitro assessments have been performed. The results indicate that N^+^-rCh-MCD forms water-soluble complexes with DAL, with a prevalent involvement of Tyr or Phe over Leu and Ala residues. The association constant of DAL with the polymeric derivative is one order of magnitude higher than that with the pristine cyclodextrin (K_a_: 2600 M^−1^ and 120 M^−1^, respectively). Additionally, N^+^-rCh-MCD shields DAL from enzymatic degradation in gastrointestinal in vitro models with a three-fold time delay, suggesting a future pharmaceutical exploitation of the polymeric derivative. Therefore, the greater affinity of N^+^-rCh-MCD for DAL and its protective effect against enzymatic hydrolysis can be attributed to the synergistic cooperation between cyclodextrin and the polymer, which is realized only when the former is covalently linked to the latter.

## 1. Introduction

Natural or synthetic polymers are widely used in pharmaceutical technology as drug delivery systems that are capable of protecting the active ingredients from possible degradation processes along the pathway to the target [1]. For this purpose, covalent conjugation or noncovalent supramolecular interactions between the drug and the controlled release platform can be exploited [2]. Moreover, polymeric materials can affect the absorption rate of active ingredients by acting on the permeability of epithelial barriers [3]. For these reasons, polysaccharides are suitable candidates, taking into account their biocompatibility, biodegradability, and relevant mucoadhesive properties. After exerting their role as drug transporters, polysaccharides are easily degraded into well-tolerated molecules by the metabolism [4]. In order to further improve their desirable properties such as drug affinity and mucoadhesivity, it is possible to modulate the physical and chemical properties of polysaccharides by modification of the constituent glycosidic units. Among amino-polysaccharides, which are made by amino-sugars monomers, chitosan (Ch) is facing fast-growing interest in the pharmaceutical industry [5,6]. It can be easily obtained by the partial deacetylation of the abundant polysaccharide chitin. Due to the high molecular weight of chitosan, and therefore a very high viscosity that hinders its use in biological applications, a first convenient modification is the degradation of chitosan, producing reduced molecular weight chitosan (rCh), which is carried out by chemical or enzymatic reactions [7]. Derivatizations of chitosan at –OH and/or –NH_2_ groups, such as in *O*- and *N*-carboxymethyl chitosan, 6-*O*-sulfate chitosan, and *N*-methylenephosphonic chitosan have been reported [8]. The quaternarization of –NH_2_ is one of the most common and relatively simple modifications, leading to a cationic polyelectrolyte that is soluble in water in a wide range of pH. Ammonium chitosan (N^+^-rCh) has also shown enhanced mucoadhesivity provided by the interactions with negative charges located on the mucous membrane [9,10,11]. To further improve its mucoadhesion properties, ammonium chitosan modified with free or *S*-protected thiol groups have been proposed [12,13,14,15,16]. Such derivatives are capable of forming covalent bonds with the cysteine-rich subdomains of mucin, which is a key component of the mucosal layers. The grafting of cyclodextrins into the chitosan backbone constitutes another promising modification, which has not yet been fully explored [17].

Cyclodextrins are cyclic oligosaccharides that are endowed with cavities that are able to encapsulate specific guests, on the basis of a convenient size fitting. The covalent conjugation of cyclodextrins seems to play a specific role in the control of releasing and protecting active ingredients, compared with the simple physical mixture of cyclodextrin/chitosan [18]. Properties of cyclodextrin–chitosan conjugates can also be affected by modification of the macrocycle counterpart, as in the use of methoxy- or ethoxy-β-cyclodextrins derivatives, the water solubility of which is remarkably enhanced with respect to parent native cyclodextrin [19,20]. In particular, a degree of substitution (DS) of 0.5 (methyl per glucose unit, i.e., an average of one methyl per two units) guarantees the maximum improvement of water solubility [19]. Aliphatic groups make the internal surface of the toroidal cavity more water-repellent, favoring the inclusion of lipophilic molecules. Methyl-cyclodextrins grafted on chitosan are hence responsible for a considerable acceleration of dissolution processes of poorly water-soluble drugs [21].

**Figure 1:**
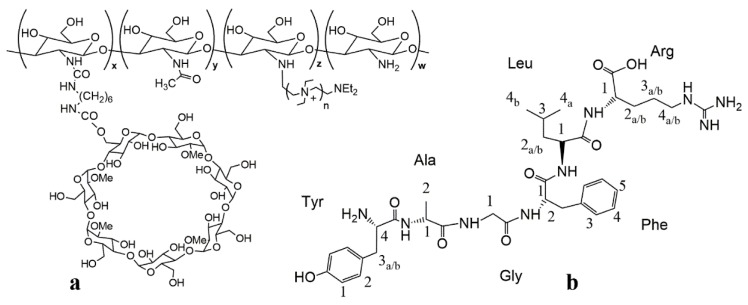
(**a**) 2-methyl-β-cyclodextrin conjugates of ammonium chitosan (N^+^-rCh-MCD) and (**b**) dalargin (DAL).

The present work is aimed to determine the role of the cyclodextrin conjugation in ammonium-chitosan grafted with 2-methyl-β-cyclodextrin (N^+^-rCh-MCD, Figure 1a) toward its binding ability to dalargin (DAL, Figure 1b), in comparison with the physical mixture of parent ammonium-chitosan and cyclodextrin (N^+^-rCh/MCD). The hexapeptide dalargin has demonstrated efficacy in the treatment of ischemia, arrhythmias, gastric ulcer, and alcohol withdrawal syndrome [22,23,24,25,26]. However, its administration is limited to i.m./e.v. injections, since it undergoes fast enzymatic degradation and a loss of efficacy within the gastrointestinal tract, similar to most of the therapeutically active peptides. Several approaches to protect peptides from enzymatic degradation have been proposed. The most used one is the loading of peptides in nanosystems such as nanoparticles, liposomes, or self-emulsifying drug delivery systems [27,28,29]. However, the realization of these nanosystems is very complicated, and their industrial application may be limited by high production costs. For this reason, we have thought of a much simpler system consisting of N^+^-rCh-MCD and DAL, which, in the aqueous physiological fluids, could give rise to the formation of a soluble macromolecular complex, in which N^+^-rCh-MCD could protect DAL from enzymatic degradation. Indeed, it is known that cyclodextrins have the ability to stabilize peptides, thus protecting them from enzymatic degradation [30]. Therefore, the protective role of the cyclodextrin in the polymer against the degradation by α-chymotrypsin (CHT) has been evaluated with respect to the separate precursors. NMR proton selective relaxation rate measurements have been exploited as a non-invasive investigation tool in order to compare dalargin to polymer affinities. UV, fluorescence techniques, and biological in vitro assessments have been also performed.

## 2. Materials and Methods

### 2.1. Materials

Chitosan was purchased from Faravelli (molecular weight = 300 kDa) (Milan, Italy). Sodium nitrite, 2-diethylaminoethyl chloride hydrochloride (DEAE-Cl∙HCl), α-chymotrypsin (CHT), dimethyl sulfoxide (DMSO), 1,6-hexamethylene diisocyanate (HMDI), and triethylamine (TEA) were purchased from Sigma-Aldrich (St. Louis, MO, USA); dalargin ((D-Ala2)-Leu-Enkephalin-Arg) was purchased from Bachem (Weil am Rhein, Germany); 2-methyl-β-cyclodextrin (DS = 0.5) was purchased from Roquette, Lestrem, France); standard pullulans (642 ÷ 6.10 kg/mol) were purchased from the American Polymers Standard Corporation (8680 Tyler Blvd., Mentor, OH, USA), and standard RC Dialysis membranes (MWCO 12500) were purchased from Spectra/Por^®^ (Sigma, Los Angeles, CA, USA). NMR deuterated solvents, D_2_O e NaOD (30%), were purchased from Deutero GmbH (Kastellaun, Germany).

HMDI and DMSO were distilled under reduced pressure (65 °C/0.2 mbar and 24 °C/0.2 mbar, respectively); TEA was refluxed over calcium hydride and distilled before use. MCD and DEAE-Cl∙HCl were anhydrified under vacuum at 37 °C.

Cell line Caco-2 was purchased from the American Type Culture Collection (ATCC^®^ HTB-37™) LGC standards, Milan, Italy) and propagated as indicated by the supplier; Minimum Essential Medium (MEM), non-essential amino acid, 0.01 M pH 7.4 Dulbecco’s Phosphate Buffer (DPBS), bovine fetal serum (BCS), glutamine, antibiotics (penicillin/streptomycin), and Hanks balanced solution were purchased from Sigma, Milan, Italy; antimycotic was supplied from Invivogen, San Diego, CA, USA. Cell proliferation reagent WST-1 was provided by Roche diagnostic, Milan, Italy.

### 2.2. Analytical Methods

GPC (Gel permeation chromatography) and HPLC analyses were carried out with a Perkin-Elmer instrument (Perkin-Elmer, Waltham, MA, USA), consisting of a Series 200 LC-290 AT pump, Rheodyne injector with a loop of 20 μL, UV-VIS SPD–6 AV detector, and Turbochrom Navigator software (Turbochrom 6.1, Perkin-Elmer, Waltham, MA, USA ). For GPC analyses, the standard pullulans were employed; the stationary phase was an X-stream H_2_O Mixed Bed 50 × 4.6 mm column, the mobile phase was acetate buffer 0.5 M (pH = 2.9), and the wavelength of the detector was set to 237 nm. For HPLC, the suitable mobile phase result was H_2_O:CH_3_CN = 70:30, the column was a C18 Aquapore OD-300 (Perkin-Elmer, Waltham, MA, USA) 7 µm 4.6 × 250 mm, and the wavelength of the detector was set to 227 nm.

NMR analyses were conducted on Varian INOVA600 spectrometer (Varian inc, Palo Alto, CA, USA) operating at 600 MHz for ^1^H and at 150 MHz for ^13^C. The samples were analyzed in a solution of D_2_O or phosphate buffer (50 mM, pH = 6.8). The temperature was controlled through Varian control unit (accuracy ±0.1 °C). 2D NMR spectra were obtained using standard sequences and the minimum spectral width in both dimensions. Two-dimensional (2D) gCOSY (gradient COrrelated SpectroscopY) maps were recorded with a relaxation interval of 1–2 s, 256–512 increments with 8–32 transients each, and 2K data points. TOCSY (TOtal Correlation SpectroscopY) maps were recorded by using a relaxation delay of 1 s, 256 increments of 4 transients, each with 2K points and a mixing time of 80 ms. 2D ROESY (Rotating-frame Overhauser Effect SpectroscopY) maps were recorded with a mixing time of 50–200 ms, relaxation delay of 1 s, 256–512 increments with 16–32 transients, and 2K data points. gHSQC (gradient Heteronuclear Single Quantum Coherence) and gHMBC (gradient Heteronuclear Multiple Bond Coherence) maps were obtained with 256 or 512 increments of 128–256 transients each and relaxation delay of 1.2 s. The gHMBC experiment was optimized for long-range J-coupling ^1^H-^13^C of 8 Hz. The mono-selective and/or bi-selective relaxation rates were measured in the approximation of the initial rate [31] through a 180° pulse set to one or two selective frequencies. After each incremental delay, a 90° non-selective pulse was applied to follow the exponential decay of the selected frequencies.

### 2.3. Preparation of Quaternary Methyl-β-Cyclodextrin Ammonium–Chitosan Conjugate

High molecular weight commercial chitosan (Ch) was depolymerized following a procedure previously reported [32]. Briefly, 5 g of Ch were dissolved in 2% acetic acid solution and reacted with NaNO_2_ (0.1 M, 12.5 mL) at 30 °C for 4 h. Afterwards, the solution pH was adjusted to pH 7.8 by using NaOH 10 M, and the obtained reduced molecular weight chitosan (rCh) was recovered by filtration, washed with water, and vacuum dried. The rCh was alkylated on -NH_2_ groups via a reaction of quaternarization optimized by Zambito et al. [2,11]: 2-methyl-β-cyclodextrin (MCD) was grafted on N^+^-rCh, following a procedure [21] articulated into several steps: 350 mg of N^+^-rCh were solubilized in 7 mL of DMSO under inert atmosphere (N_2_) overnight. Sequentially, N^+^-rCh (350 mg) and TEA (0.056 mL, 0.40 mol) were added to a solution of HMDI (0.7 mL, 4.4 mmol) in DMSO (8 mL), and the mixture was placed in an oil bath at 70 °C, under inert atmosphere. The reaction was maintained for 2 h and controlled steadily, until it became turbid. Thereafter, the polymer was precipitated in cold diethyl ether (0 °C) and washed thrice, eliminating the supernatant, and then redissolved in 3 mL of DMSO. MCD (2.38 g in 7 mL of DMSO) and 0.140 mL of TEA were directly used to dissolve the recovered ammonium–chitosan grafted with HMDI spacers; afterwards, the mixture was heated at 70 °C for 3 h. Finally, the solution was quenched by adding it to deionized water at 80 °C, drop by drop, under stirring, showing an initial effervescence. After 1 h, the mixture was dialyzed with deionized water for 3 days. The insoluble portion of product was removed through centrifugation (Sorvall, MTX, 20.000 rpm, 30 min, 4 °C, thermo Fisher Scientific In, Waltham, MA, USA) and the supernatant portion was dried, giving the cyclodextrin–polymer conjugate (N^+^-rCh-MCD).

### 2.4. Characterization of DAL Inclusion Complexes

#### 2.4.1. Complexes Stoichiometry (Job’s Plot)

The complex stoichiometry of MCD and DAL, and polymers and DAL, were evaluated by the continuous variation method (Job’s plot). Series of samples were prepared keeping constant the total molar concentration (275 μM) of the binding partners (DAL and MCD), but varying their mole fraction between 0 and 1. Similarly, complex stoichiometry was evaluated for MCD in the presence of N^+^-rCh and for the MCD-grafted polymer (N^+^-rCh-MCD); the dilution specifications are reported in Table S1 of the supplementary material). The absorbance of the solution sets was acquired by UV-VIS (Perkin Elmer Precisely lambda-25) at time 0 min, 1 h, and 24 h from solution mixing, with solutions kept at room temperature. The second derivative calculation was performed in order to lessen the polymers’ nonspecific contribution. Job’s plot was built on the difference between 275 and 316 nm absorbance values (ΔA). The plot displays the variation of ΔA∙χ vs χ with χ = [DAL]/([DAL] + [MCD]). The abscissa value corresponding to the curve maximum represents the complex stoichiometry as 1:2, 1:1, or 2:1 with χ = 0.33, χ = 0.5, or χ = 0.66, respectively.

#### 2.4.2. Evaluation of Complex Association Constant (Benesi–Hildebrand Method)

Complex association constant (K_a_) was determined by spectrometric titration based on the Benesi–Hildebrand method [33] and performed both by UV-VIS and fluorescence spectrometry. Two sets of dilutions were prepared (Tables S2–S5, Supplementary material). In the case of UV spectrometry, 275 μM DAL was kept, whereas MCD concentrations varied, either as MCD alone or as conjugated (N^+^-rCh-MCD). The solutions were prepared and analyzed after being equilibrated for 1 h at room temperature. UV spectra in the range 220–325 nm were collected, and the intensity of absorbance at 282 nm was normalized on the correspondent value at 247 nm. Diversely, for fluorescence spectroscopy evaluations, DAL concentration was kept at 70 μM, and samples were analyzed by using a PerkinElmer LS 45 fluorimeter, with excitation λ set at 270 nm and the emission spectrum collected between 340 and 270 nm. The variation of fluorescence intensity was evaluated at 304 nm, and it was normalized on 285 nm.

K_a_ was calculated from the ratio between the intercept and angular coefficient of the linear regression of a modified Benesi–Hildebrand equation (Equation (1)) [34]:

1/(I − I_0_) = 1/{(I′ − I_0_) K_a_} 1/[MCD] + 1/(I′ − I_0_) (1)

where [MCD] and [DAL] referring to the total amount of MCD and DAL in solution, I and I_0_ are the intensities in the presence and absence of MCD, respectively, and I’ is the value when the DAL molecules are all complexed with MDC.

### 2.5. Stability under Enzymatic Hydrolyses

Stock solutions of dalargin (1 mg/mL) either alone or as a 1:1 complex with MCD or polymers were mixed with α-chymotrypsin (0.36 mg/mL) solutions to obtain 0.50 mg/mL and 0.18 mg/mL (corresponding to 7.2 UI/mL) final concentrations, respectively [35]. All solutions were prepared in phosphate buffer (pH = 6.8, 0.4 M), and the enzymatic hydrolysis was performed under thermostated conditions (37 °C). All samples were analyzed after 5 min by interrupting the digestion with temperature shock (4 °C) and extensive dilution (1:20 with cold HPLC mobile phase). Residual DAL was expressed as the percentage of initial DAL as detected by the HPLC method.

DAL complexes were also prepared as solid powder by freeze-drying in a VirTis AdVantage wizard 2.0, SP Scientific, lyophilizator. Samples were frozen at a temperature of −40 °C and sublimated at a pressure of 30–40 mTorr, with an end point at 16 °C. The lyophilized samples containing DAL either alone or in a 1:1 complex were redispersed in PB and submitted to α-chymotrypsin hydrolysis, as already described. 

### 2.6. Sample Preparation for NMR Studies

#### 2.6.1. Affinity Studies 

In the preparation of samples for the NMR analyses, the use of standardized solubilization procedures is crucial, as pointed out in our precedent work [12]. As a matter of fact, the conformational stabilization phenomena of polymeric material can occur over time, especially for high molecular weight polymers, thus affecting the NMR relaxation parameters. For these reasons, the relaxation rate measurements were repeated over time in order to avoid time-dependent effects on NMR parameters. DAL concentration was fixed at 0.50 mg/mL (the same concentration was used in HPLC degradation studies). On the basis of the knowledge of cyclodextrin content in the conjugated polymer, an N^+^-rCh-MCD concentration of 3.60 mg/mL (corresponding to 2.50 mg of polymer into the NMR tube) was selected in order to set the 1:1 grafted MCD/DAL molar ratio. Accordingly, the ammonium–chitosan precursor concentration was 2.00 mg/mL (corresponding to 1.40 mg polymer into the NMR tube).

For NMR analyses, all polymers were dissolved in D_2_O at 40 °C in vortex (600 rpm) for 2 h. After 1 h without stirring at room temperature, a solution of DAL was added to obtain the final ratio of 2.50 mg/0.33 mg for the mixture N^+^-rCh-MCD/DAL or 1.40 mg/0.33 mg for the mixture N^+^-rCh/DAL into the NMR tube. The corresponding mixture was kept at 25 °C for 2 h in vortex and then for another 2 h motionless before NMR measurements. The DAL/MCD mixture was kept under stirring at room temperature for 2 h and then kept for another further 2 h without stirring.

#### 2.6.2. Enzymatic Hydrolyses

The same DAL/enzyme ratio and concentrations of components selected for HPLC were used in NMR analysis at 37 °C through the entire digestion process. DAL and α-chymotrypsin stock solutions were prepared in phosphate buffer (pH = 6.8) and mixed directly into the NMR tube containing predissolved polymers at 40 °C for 2 h. 

### 2.7. Biological Evaluation 

#### 2.7.1. Cell Viability

Cell viability in the presence of either solutions of DAL or solutions of DAL and polymers/cyclodextrin was carried out using the human cell line Caco-2. Cells were grown in Minimum Essential Medium (MEM) supplemented with 1% (*v/v*) non-essential amino acid in 10% (*v/v*) fetal bovine serum, 2 mM glutamine, 100 U/mL penicillin, 100 g/mL of streptomicyn, and 0.2% (*v/v*) antimycotic. A subconfluent monolayer of Caco-2 cells was trypsinized using a 0.25% trypsin and 1 mM EDTA solution, centrifuged at 1000 rpm for 5 min, resuspended in growth medium, and counted. Caco-2 cells were seeded in each well of 96-well plates at a seeding density of 2 × 10^4^ cells/well. Cells were incubated at 37 °C, 5% CO_2_ for 24 h until 60%–70% confluence was reached. Then, the medium was then removed from each well, washed with Hanks balanced solution (HBSS), and replaced with HBSS containing the samples DAL, DAL/N^+^-rCh-MCD, DAL/N^+^-rCh, DAL/MCD, and DAL/N^+^-rCh/MCD, all containing same amount of DAL (in the range 15–150 μg/mL) and prepared maintaining a 1:1 complex stoichiometry. After 3 h of incubation, DAL containing media were removed and substituted by WST-1 reagent solution (according to manufacturer instructions), for 4 h at 37 °C, 5% CO_2_. Afterwards, formazan dye absorbance was quantified at 450 nm with the reference wavelength at 655 by using an Enspire 230 (Perkin-Elmer, Waltham, MA , USA) multilabel reader.

#### 2.7.2. In Vitro Evaluation of Protection from Enzymatic Degradation

A Caco-2 monolayer was obtained by cell seeding (10^5^ cells/well) on a polycarbonate membrane filter (pore size 0.4 μm, area 1.12 cm^2^) in Corning Transwell, 12-well plates (Sigma-Aldrich, Saint Louis, MO, USA). Thereafter, 0.5 and 1.5 mL of culture medium was added to apical and basolateral compartments of the transwell inserts, respectively; the culture medium was replaced every two days for 24 days. As a rule, the experiments were performed with Caco-2 monolayers that have been grown on filter inserts for 21–29 days. By day 21, the monolayers have become differentiated with regard to the expression of many transport proteins, enzymes, and brush border hydrolases [36]. The TransEpithelial Electrical Resistance (TEER, Ωcm^2^) across the Caco-2 monolayer was determined by measuring the potential difference between the apical and basolateral sides of the transwell using the Millicell-ERS (Millipore, Milano, Italy). TEER was measured prior to each experiment to ensure the confluence of the monolayers, during transport studies (every 30 min), and at 24 h from the samples’ removal to assess monolayer stability recovery. Experiments were performed by using DAL-containing samples, which had either DAL alone or the presence of polymers and cyclodextrin. Caco-2 monolayers were gently rinsed twice with prewarmed HBSS and preincubated at 37 °C for 15 min with 0.5 mL and 1.5 mL of HBSS for the apical and basolateral side, respectively. Afterwards, the sample solutions (0.5 mL), all containing DAL 50 μg/mL, were applied to the apical side of each of the transwell inserts. At the end of the experiment (3 h), the apical chambers media were collected and analyzed by HPLC for DAL content evaluation.

## 3. Results and Discussion

### 3.1. NMR Characterization 

Five regions were distinguished in the ^1^H NMR spectrum of ammonium–chitosan N^+^-rCh (Figure S1, Supplementary material). A quantitative NMR protocol developed for these specific systems [2] was applied in order to determine the degree of acetylation (8.8%), degree of derivatization (42.4%), and the ratio between the quaternary and neutral nitrogen of modified chains (n = 2.3). 

Through GPC analysis, the molecular weights of the precursor (rCh, 134 kg/mol) and ammonium–chitosan (N^+^-rCh, 184 kg/mol) were determined. Moreover, knowledge of the degree of acetylation and of the molecular weight of rCh made it possible to estimate the average number of units (800) in N^+^-rCh.

**Figure 2:**
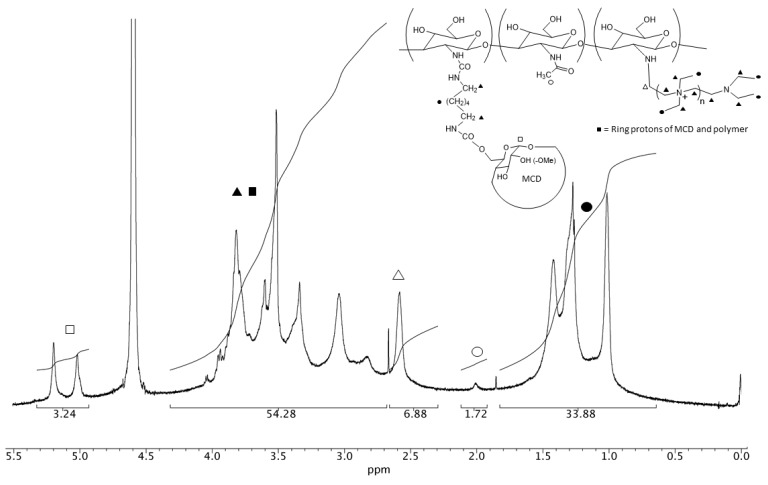
^1^H NMR spectrum (600 MHz, D_2_O, 37 °C) of N^+^-rCh-MCD (3.7 mg/mL). Black and white dots, squares and triangles refer to the corresponding protons marked on the structure.

The ^1^H NMR spectrum of cyclodextrin-grafted ammonium–chitosan polymer (N^+^-rCh-MCD) (Figure 2) roughly corresponds to the superimposition of N^+^-rCh and MCD spectra. The degree of methylation of the cyclodextrin (DS = 0.5) was checked by NMR analysis of MCD (Figure S2, supplementary material). The use of a refined NMR quantitative analysis protocol (supplementary material) of N^+^-rCh-MCD led to establishing that the N^+^-rCh-MCD polymer contains 22% *w/w* of MCD and 18% *w/w* of spacer. Although the percentages of MCD and HMDI are almost equal, the conspicuous difference in their MWs indicates that there are numerous spacer chains bonded to the polymer only but unbound to the cyclodextrin. Excluding cross-linking phenomena, it was possible to estimate the minimum molecular weight of the conjugate, knowing the percentage by weight of the precursor and, therefore, the moles of conjugate, reporting a MW of 306 kg/mol, with 331 units of HMDI and 57 MCD, which was testimony to the fact that there is so much free spacer. DAL (Figure 1b) was characterized by ^1^H e ^13^C NMR spectroscopy (Figures S3–S5, Tables S6 and S7, supplementary material).

### 3.2. Inclusion Complexes

#### 3.2.1. Complexes Stoichiometry and Association Constant

The complex formation between DAL and MCD was investigated and compared to that of DAL with MCD conjugated to the quaternary ammonium chitosan (N^+^-rCh-MCD) or in the presence of plain N^+^-rCh (N^+^-rCh / MCD). In all cases, the χ value was 0.5, corresponding to the 1:1 complex stoichiometry of DAL/MCD (Table S8, Supplementary material). The results evidenced that the quaternary ammonium polymer was not interfering with the formation of the complex neither when dissolved together with MCD nor when covalently linked to MCD. Additionally, χ values were not affected by the incubation time, indicating a prompt complexation between MCD and DAL (Table S8, supplementary material). 

The determination of the association constant (K_a_) for complexes of DAL/MCD and of DAL/N^+^-rCh-MCD was performed both by UV and fluorescence spectrometry. Both techniques exploited the gradual variation of DAL absorbance or fluorescence in the presence of increasing amounts of host agent. These variations were better monitored by using DAL fluorescence, whereas UV spectrometry was poorly applicable to DAL/MCD complexes, which would have required an excessive amount of the binding partners to reveal the absorbance variation. Differently, this latter technique was successfully used only for the DAL/N^+^-rCh-MCD complex. The calculated K_a_ values are reported in Table 1. 

The good linearity of data regression confirmed the formation of a 1:1 complex in both cases, DAL/MCD and DAL/N^+^-rCh-MCD. More importantly, the K_a_ evaluated for the peptide complexed to the N^+^-rCh-MCD polymer was one order of magnitude higher than that calculated for MCD alone. Despite the two different spectroscopic techniques, the values are consistent with each other. 

**Figure 3:**
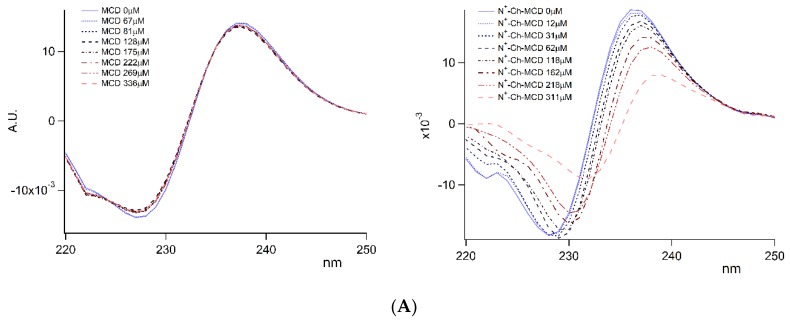

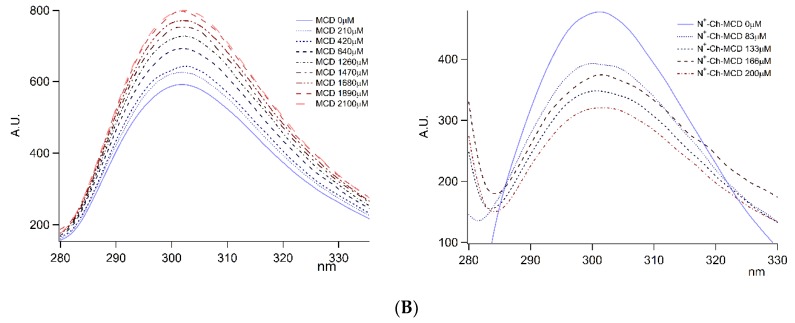
Complex titration by UV and fluorescence spectrometry. (**A**) Overlay of UV second derivative spectra; (**B**) Overlay of emission spectra collected by excitation at λ 275 nm.

It is worth mentioning that the spectrometric titration performed for the evaluation of K_a_ evidenced a clear variation of DAL absorbance peak at 227 nm in terms of intensity and wavelength, as clearly displayed by the overlay of the II derivative absorbance spectra of DAL/N^+^-rCh-MCD complexes (Figure 3A). Similarly, the greatest fluorescence variation of complexed DAL was revealed at the 304 nm emission band (Figure 3B). Both phenomena can be related to a main variation of the chemical environment surrounding Tyr residue, which appeared more pronounced for the DAL/N^+^-rCh-MCD complex. As a matter of fact, the Tyr ABS peak at 227 nm is typically exploited for protein unfolding studies [37], and Tyr fluorescence is predominant with λ^ex^ 275 and λ^em^ peaks at 304 nm [38].

#### 3.2.2. NMR Interaction Studies and Complex Stereochemistry

The interaction between a small molecule (G) and a macromolecule (H) can be described by the simple equilibrium reported in Equation (2). In fast exchange conditions, the observable NMR parameters (P_obs_) represent the weighted average between the bound (P_b_) and free (P_f_) states (Equation (3)).

H + G ⇌ HG(2)

P_obs_ = χ_f_ P_f_ + χ_b_ P_b_ (3)

where χ_f_ and χ_b_ are the molar fractions of the ligand in the free and bound states, respectively. By virtue of the marked difference between the molecular weights of H and G, it is necessary to operate with a large excess of G with respect to H in order to obtain an observable NMR signal. In these conditions, the molar fraction of the small molecule in its complexed form (χ_b_) is negligible. Therefore, the choice of sensitive parameters is required to detect significant changes in the bound state with respect to the free state. Longitudinal relaxation rates represent an effective tool in the analysis of the interaction between small molecules and macromolecules. In particular, proton mono-selective relaxation rates (R_i_^ms^ = 1/T_i_^ms^), measured by following the recovery of the magnetization of the selectively inverted spin *i*, are remarkably more sensitive than non-selective ones [39]. Considerable increases of R^ms^ are expected when the small molecule undergoes a change in molecular dynamic from fast (*ω^2^τ_c_^2^* ≪ 0.6, where ω is the Larmor frequency and *τ_c_* is the rotational correlation time) to slow motion regimes (*ω^2^τ_c_^2^* ≫ 0.6), which is typical when bounded to a macromolecule. The cross-relaxation term (*σ*_ij_), referring to the spin *i* and *j* at *r*_ij_ distance, is another susceptible parameter to complexation phenomena. *σ*_ij_ can be experimentally obtained from the difference between the bi-selective relaxation rate (detected by following the recovery of the magnetization of the nucleus *i* in conditions of simultaneous inversion of the spin *j*, R_ij_^bs^ = 1/ T_ij_^bs^) and mono-selective relaxation rate, as shown in Equation (4).

σ_ij_ = R_ij_^bs^ – R_i_^ms^ (4)

In the two limit cases of fast and slow -motion regimes, the expression of *σ_ij_* can be calculated by Equations (5) and (6), respectively.

σ_ij_ = 0.5 γ^4^ ħ^2^ r_ij_^−6^ τ_c_  (ω^2^τ_c_^2^ ≪ 0.6) (5)

σ_ij_ = −0.1 γ^4^ ħ^2^ r_ij_^−6^ τ_c_  (ω^2^τ_c_^2^ ≫ 0.6) (6)

Therefore, a negative σ indicates a slowdown of molecular motion, which is generated by the interaction of the active ingredient with the macromolecule.

Due to the extensive superimposition between DAL and the polymers’ resonances, only the selective relaxation rates of phenylalanine and tyrosine aromatic protons and methyl moieties of leucine of DAL (0.5 mg/mL) were measured in solutions containing pure DAL or its mixtures with polymers/cyclodextrin. 

The mono-selective and bi-selective relaxation of DAL protons is collected in Table 2, along with selected cross-relaxation terms. NMR measurements were performed both immediately after preparation of the solutions and after one week, with negligible variations in the relaxation parameters recorded.

The presence of an equimolar amount of MCD did not produce any significant variations in the relaxation parameters of DAL (Table 2), indicating minor drug/macrocycle interactions, according to the very low amount of bound molar fraction (7%) at the concentration of 0.68 mM. The same trend was found for the binary mixture DAL/N^+^-rCh, with negligible variations with respect to free DAL. Importantly, the minimum impact of the presence of N^+^-rCh also rules out possible interfering effects on relaxation parameters attributable to viscosity changes caused by the presence of the polymer, rather than to chemical interactions with the macromolecule. On the contrary, outstanding increments in relaxation rates were recorded when dalargin is mixed with ammonium–chitosan covalently conjugated with MCD (DAL/N+-rCh-MCD), especially for aromatic moieties. As an example, R_1_^ms^ of H_3_^Phe^ increases from 0.62 s^−1^ to a value of 4.54 s^−1^. Cross-relaxation rates respectively decrease to the values of -0.88 s^−1^ and -0.62 s^−1^ for H_3_^Phe^/H_4_^Phe^ and H_1_^Tyr^/H_2_^Tyr^ proton pairs, due to the relevant slowing down of the molecular motion of DAL consequent to its interaction with N+-rCh-MCD. Interestingly, the simple physical mixture of ammonium–chitosan and cyclodextrin (DAL/N^+^-rCh/MCD) produced negligible variations of the relaxation parameters compared to pure DAL, confirming the key role of the covalent grafting of cyclodextrin to the polymer (Table 1).

To obtain a parameter that reflects the different degree of involvement of DAL fragments in the interaction with polymers, normalized relaxation rates were calculated [|R| = (R_1_^mix^ − R_1_^free^)/R_1_^free^], showing a major involvement of tyrosine and phenylalanine group (Table 3). The particularly high value for the *ortho*-protons to hydroxyl group of tyrosine is due to the role of the polar group in the interaction with the polysaccharide material.

**Figure 4:**
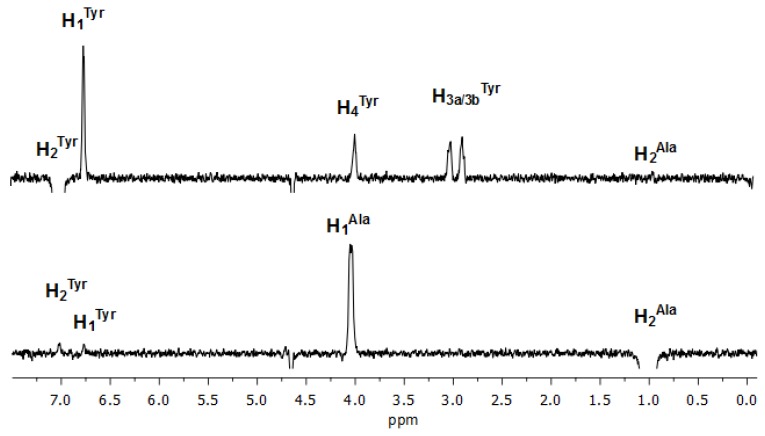
One-dimensional (1D) Rotating-frame Overhauser Effect SpectroscopY (ROESY) spectra (600 MHz, D_2_O, 25 °C) of H_2_^Tyr^ and H_2_^Ala^ of DAL (9.8 mM).

The mixture DAL/MCD was employed as a model for investigating the nature of the interaction of DAL with MCD. The study was carried out at 9.8 mM concentration, which guaranteed a significant bound molar fraction of DAL (about 40%) on the basis of the value of the association constant DAL/CD (120 M^−1^). Such a kind of investigation was not possible for the mixture DAL/N^+^-rCh-MCD due to the extensive overlapping of polymer and cyclodextrin signals. An extended conformation of DAL was found on the basis of the analysis of 1D ROESY spectra, where any significant dipolar interactions were not detected between protons bearing different amino acidic residues. As an example, the selective perturbation of Tyr protons produces negligible ROE effects on the adjacent Ala protons (Figure 4).

In order to point out the nature of the DAL groups that were more involved in the interaction to MCD, complexation shifts were calculated (|Δδ| = |δ^DAL/MCD^ – δ^MCD^|, Hz). For this type of analysis, TOCSY traces were accurately analyzed: starting from the anomeric protons H_1_ and H_1′_ of MCD, resonances of the corresponding glucosidic rings protons were individuated. Complexation shifts were remarkably higher for H_3_/H_3′_ and H_5_/H_5_, which were located inside the hydrophobic cavity of MCD with a deep inclusion from the edge of larger diameter and supported by considerable values of Δδ for H_5_/H_5′_ (Table 4).

**Figure 5:**
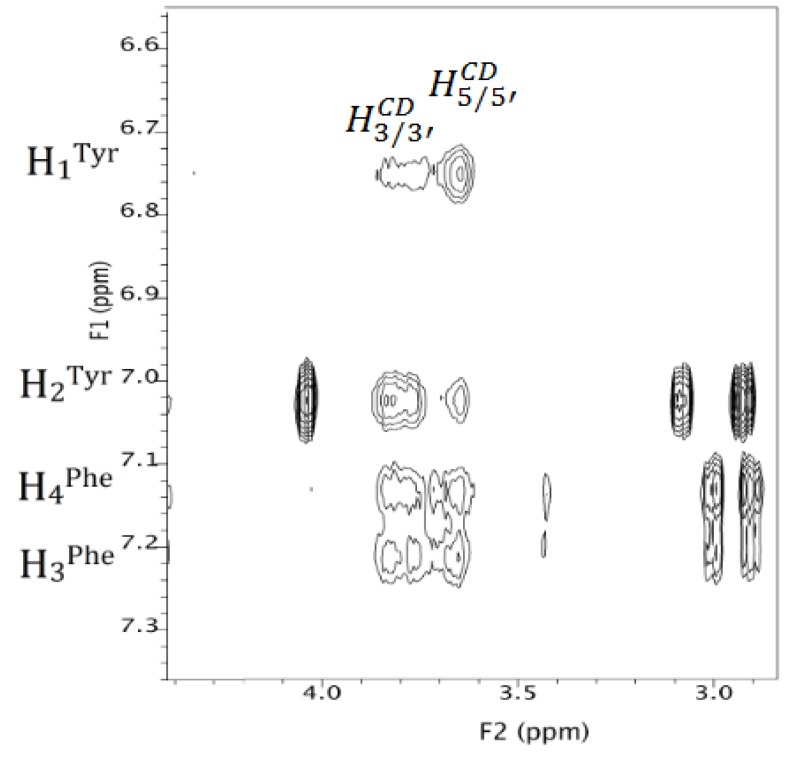
ROESY map (600 MHz, D_2_O, 25 °C, 9.8 mM) of DAL/MCD mixture between 6.6–7.3 ppm and 2.8–4.4 ppm.

Detailed information on the stereochemistry of the inclusion were obtained from the ROESY map, (Figure 5 and Figure S6, supplementary material), showing ROE effects mainly between the aromatic protons of DAL and the H_3_/H_3′_ and H_5_/H_5′_ internal protons of the cyclodextrin.

In particular, H_1_^Tyr^ produces much more intense ROE effects on the H_5_/H_5′_ (narrower rim) compared with the H_3_/H_3′_ (wider rim), while H_2_^Tyr^ showed an opposite trend, indicating that the phenolic group is embedded in the cavity of the cyclodextrin deeply and confirming what was observed with UV and fluorescence spectrometry.

Phenylalanine is also widely involved in the interaction with cyclodextrin, but the effects produced at H_3_/H_3′_ and H_5_/H_5′_ frequencies have comparable intensities, which is probably because of aromatic moieties mobility within the cavity of the cyclodextrin.

It is noteworthy that both methyl protons of leucine and alanine originated dipolar interactions with the internal H_3_/H_3′_ protons and external methoxy protons of the cyclodextrin, thus revealing superficial interactions at the wide rim of the cyclodextrin.

Therefore, taking into account that 1 to 1 complexation stoichiometry has been demonstrated, it can be concluded that dalargin may originate multiple interactions with the cyclodextrin, involving the deep inclusion of the aromatic groups of tyrosine or phenylalanine, as well as superficial interactions of the alkyl groups of leucine or alanine at the wide rim of the cyclodextrin.

### 3.3. Kinetic Studies of Dalargin Enzymatic Hydrolysis

The protective role of N^+^-rCh-MCD against DAL enzymatic hydrolysis was studied with two orthogonal methods (HPLC and NMR). α-Chymotrypsin (CHT) was chosen as the model enzyme for study of the degradation of DAL in the human body [35]. CHT belongs to the proteases and is capable of breaking peptide bonds with preference for tyrosine, phenylalanine, and leucine residues [40]. Pure DAL is stable in water at 37 °C for several days, as demonstrated by comparing ^1^H NMR spectra recorded at different time after preparation: no differences were recorded neither in chemical shifts nor in the peaks’ integrated areas.

**Figure 6:**
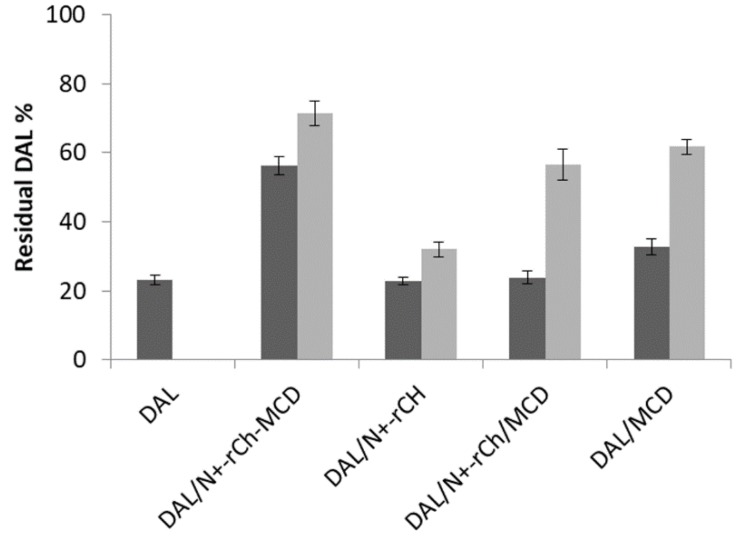
α-chymotrypsin (CHT) hydrolysis of DAL: Residual DAL % after 5 min of CHT activity on solutions (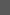); on lyophilized complexes (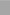). Error bars indicate the SD values of three independent experiments.

The digestion of DAL by CHT was performed under controlled conditions to simulate the digestion occurring in the intestinal environment. A first characterization was performed on freshly prepared solutions of DAL, either alone or mixed with MCD, N^+^-rCh /MCD, or N^+^-rCh-MCD. The analysis that was carried out 5 min after the addition of the enzyme evidenced that the peptide underwent a rapid degradation, with a reduction equal to 80% of the initial content. The degradation process was merely slowed down in the presence of MCD, in an equal extent of MCD and MCD mixed with N^+^-rCh. Diversely, when using the MCD-grafted polymer (N^+^-rCh-MCD), DAL suffers only a 45% reduction. The results highlighted that only when MCD is covalently bound to the quaternary polymer was there a significant protection from the enzymatic attack (Figure 6).

A similar digestion was conducted also on lyophilized samples that were freshly redispersed in buffer before adding the enzyme. Similarly, plain DAL was rapidly degraded, but an extended stability was observed for the DAL/MCD complex and even more for the complex DAL/N^+^-rCh-MCD (Figure 4). Therefore, it was assumed that the conjugate was able to protect DAL from enzymatic digestion, and that the lyophilization process strengthened the interaction of DAL with the cyclodextrin, increasing its protection [41]. Despite the augmented stability due to the lyophilization process, the use of the conjugated polymer N^+^-rCh-MCD led again to a significantly reduced digestion compared to the use of N^+^-rCh/MCD mixture (*p* < 0.01).

**Figure 7:**
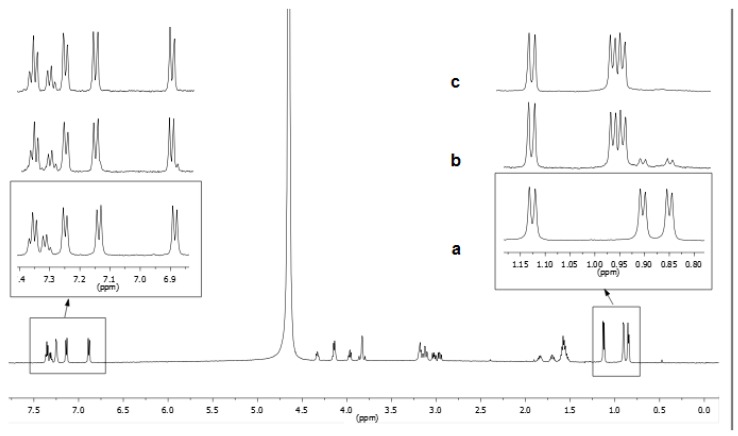
^1^H NMR spectra (600 MHz, D_2_O, phosphate buffer, pH = 6.8, 37 °C) of pure DAL (0.68 mM, (**a**) and of the DAL/CHT after 5 min (**b**) and 14 min (**c**).

By using the same DAL/CHT molar ratio selected for HPLC analysis, low and high frequency NMR spectral regions of dalargin, pure (Figure 7a) and in presence of CHT (Figure 7b, c), were monitored over time at 37 °C. In the binary mixture DAL/CHT, a rapid decrease of the original dalargin signals was observed already after 5 min with the simultaneous formation of new peaks, which was attributable to the digestion products. A comparison of integrated areas showed that the digestion was almost complete after 14 min. In order to obtain a quantitative parameter, we refer to the lifetime of the drug (*t_f_*), the specific time in which non-degraded DAL reached 6% with respect to the hydrolyzed products. If the mixture is maintained at room temperature (25 °C), the *t_f_* of DAL is extended up to 45 min, owing to a reduced activity of the enzyme.

Effects due to the presence of MCD and/or the modified chitosans on the rates of hydrolysis were evaluated: as reported in Table 5, MCD, N^+^-rCh, or their physical mixture MCD/N^+^-rCh only slightly affected the hydrolysis rate. On the contrary, conjugated chitosan N^+^-rCh-MCD produced remarkably prolonged *t_f_*, confirming what was observed at a shorter digestion time with the HPLC technique. It clearly appeared that the covalent binding of MCD to the chitosan backbone was essential to generate a protecting effect against the DAL hydrolytic pathways.

### 3.4. Biological Evaluation

**Figure 8:**
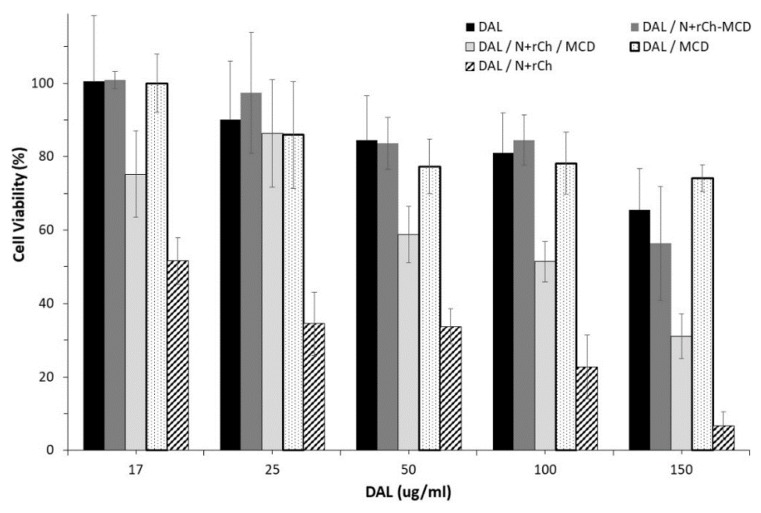
Caco-2 cells cell viability assay. Error bars indicating SD values of eight replicates.

A quantitative evaluation of cell viability performed by WST-1 assay on a Caco-2 cell line (Figure 8) indicated a high tolerability for the complex of DAL with N^+^-rCh-MCD, which was comparable to what was observed for plain DAL and DAL complexed with MCD. Differently, N^+^-rCh polymer negatively affected cell viability, reducing it drastically at the tested concentration. Such behavior was shielded by the presence of MCD, especially when covalently conjugated as for the polymer N^+^-rCh-MCD.

**Figure 9:**
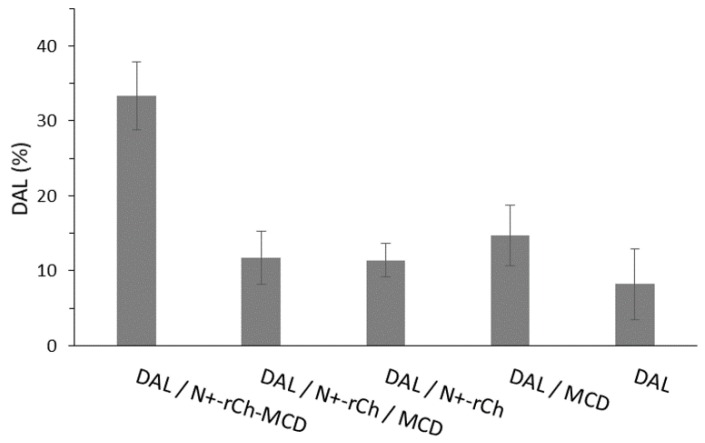
Percentage of DAL remaining in the apical chamber after 3 h of incubation on Caco-2 monolayers. Error bars indicate the SD values of three experiments.

DAL stability toward enzymatic degradation in the presence of N^+^-rCh-MCD polymer significantly increased when tested on Caco-2 monolayers (Figure 9). After 3 h of incubation, DAL contents in the apical chamber of plain DAL, DAL/MCD, DAL/N^+^-rCh, and DAL/N^+^-rCh/MCD physical mixtures were not significantly different (in the range of 4.1–7.3 μg/mL, corresponding to 8%–15% of residual DAL). The MCD-bearing quaternary polymer (N^+^-rCh-MCD) exhibited 16.7 μg/mL of DAL after 3 h of incubation, confirming its stronger stabilizing effect also in a more complex biological environment.

The effect of cyclodextrin and polymer derivatives on monolayers permeability was monitored by TEER measurements. TEER values exhibited the same trend of the untreated control layer until 1 h of incubation, followed by a plateau in the range of 86%–100% for the remaining contact time. On the contrary, N^+^-rCh determined a significant reduction of TEER values after 2–3 h, reaching 64%. This finding is in agreement with our previous research studies [16], stressing that quaternary ammonium chitosan acts as a loosening/opening agent for intercellular tight junctions, resulting in the increased paracellular permeability of the monolayers, with the reversibility of the effect (Figure S7, supplementary material).

## 4. Conclusions

MCD grafted to quaternary ammonium chitosan (N^+^-rCh-MCD) has proved to be effective as a host macromolecule for the short, labile, and therapeutically active peptide DAL. This finding, along with the previously performed investigations [21,42], suggests the suitability of MCD-grafted quaternary chitosans as functional polymers for the oral administration of poorly bioavailable actives.

It has been demonstrated that the interaction of dalargin with MCD occurs through a deep inclusion in the oligosaccharide cavity, involving mainly Tyr or Phe aromatic groups or, with minor extent, the alkyl groups of the Leu and Ala units. Additionally, the covalent binding of MCD to the mucoadhesive chitosan derivative is not hampering the complexing ability of MCD, but rather further increasing the interaction with dalargin. The MCD grafting has a positive effect on polymer cytocompatibility, and it has been demonstrated that the polymer behaves as a shield versus the proteolytic attack of intestinal enzymes only when covalently linked to the MCD.

The mentioned features were described for water-soluble complexes of N^+^-rCh-MCD/DAL, but the preliminary evaluation of lyophilized samples showed improved stabilization of the peptide, indicating the perspective of using a processed powder for the preparation of solid dosage forms.

## Figures and Tables

**Table 1:** Association constant (K_a_) determined by UV absorbance or fluorescence spectrometry, for DAL complexing with 2-methyl-β-cyclodextrin (MCD) or N^+^-rCh-MCD. Standard deviation (SD) and the coefficient of determination (R^2^) of the linear regression are also displayed.

	Absorbance			Fluorescence		
	K_a_ (M^−1^)	SD	R^2^	K_a_ (M^−1^)	SD	R^2^
DAL/MCD	-	-	-	120	10	0.999
DAL/N^+^-rCh-MCD	2326	406	0.999	2617	307	0.990

**Table 2:** Mono-selective relaxation rates (*R^ms^*, s^−1^), bi-selective relaxation rates (*R^bs^*, s^−1^), and cross-relaxation terms (*σ*, s^−1^) of the proton pair H_3_/H_4_^Phe^ and H_1_/H_2_^Tyr^ of DAL (600 MHz, 25 °C, D_2_O, 0.68 mM).

	δ (ppm)	R_1_^ms^ (s^−1^)				
		DAL	DAL/MCD	DAL/N^+^-rCh	DAL/N^+^-rCh-MCD	DAL/MCD/N^+^-rCh
H_3_^Phe^	7.11	0.62	0.59	0.61	4.54	0.60
H_4_^Phe^	7.22	0.54	0.54	0.52	4.35	0.50
H_5_^Phe^	7.18	0.66	0.57	0.61	5.00	0.53
H_1_^Tyr^	6.77	0.36	0.32	0.34	4.54	0.34
H_2_^Tyr^	7.02	0.58	0.57	0.55	4.54	0.54
H_4a_^Leu^	0.78	1.69	1.65	1.67	4.17	1.67
H_4b_^Leu^	0.72	1.59	1.58	1.59	4.17	1.59
		R_1_^bs^ (s^−1^)				
H_3_^Phe^	7.11	0.63	0.60	0.62	3.66	0.62
H_4_^Phe^	7.22	0.55	0.55	0.54	3.47	0.53
H_1_^Tyr^	6.77	0.39	0.35	0.36	3.92	0.37
H_2_^Tyr^	7.02	0.61	0.60	0.57	3.92	0.57
		*σ* (s^−1^)				
H_3_/H_4_^Phe^	-	0.01	0.01	0.01	−0.88	0.02
H_1_/H_2_^Tyr^	-	0.03	0.03	0.02	−0.62	0.03

**Table 3:** Normalized relaxation rates (|R| = (R_1_^mix^ − R_1_^free^)/R_1_^free^) of selected protons of DAL in the binary mixture DAL/N^+^-rCh-MCD.

	DAL/N^+^-rCh-MCD						
	H_3_^Phe^	H_4_^Phe^	H_5_^Phe^	H_1_^Tyr^	H_2_^Tyr^	H_4a_^Leu^	H_4b_^Leu^
|R|	6.32	7.06	6.58	11.6	6.83	1.47	1.62

**Table 4:** MCD (600 MHz, D_2_O, 25 °C, 9.8 mM) complexation shifts.

MCD	|Δδ| (Hz)	MCD	|Δδ| (Hz)
H_1_	9.5	H_4_	4.2
H_1′_	4.1	H_4′_	1.2
H_2_	4.5	H_5_	41.8
H_2′_	6.1	H_5′_	49.2
H_3_	27.6	H_6_/H_6′_	3.2
H_3′_	25.3		

**Table 5:** Complete hydrolyses times (*t*_f_, min) of DAL (0.68 mM) in fresh mixtures and lyophilized mixture in the presence of CHT, as recorded by ^1^H NMR (600 MHz, 37 °C, phosphate buffer, pH = 6.8).

Mixture	*t*_f_ (min)
DAL/CHT/MCD	16
DAL/CHT/N^+^-rCh	18
DAL/CHT/N^+^-rCh/MCD	17
DAL/CHT/N^+^-rCh-MCD	61

## Supplementary Materials

Available online at https://www.mdpi.com/2073-4360/12/2/474/s1.

## Abbreviations

BCS	bovine fetal serum
Ch	chitosan
CHT	α-chymotrypsin
DAL	dalargin
DEAE-Cl HCl	2 diethylaminoethyl chloride hydrochloride
DMSO	dimethyl sulfoxide
DPBS	Dulbecco’s phosphate buffer
HMDI	1,6-hexamethylene diisocyanate
MCD	2-methyl-β-cyclodextrin
MEM	minimum essential medium
N^+^-rCh	reduced molecular weight ammonium chitosan
N^+^-rCh-MCD	ammonium-chitosan grafted with 2-methyl-β-cyclodextrin
rCh	reduced molecular weight chitosan
TEA	triethylamine
