# Supplementary material for: Improvement of Peptide Affinity and Stability by Complexing to Cyclodextrin-Grafted Ammonium Chitosan
Source: Polymers (Basel). 2020 Feb 19;12(2):474. doi: 10.3390/polym12020474 (PMC7077720; doi:10.3390/polym12020474)
Supplement: Supplementary file 1 [file polymers-12-00474-s001.pdf]

polymers

Supplementary material

Improvement of peptide affinity and stability by complexing to cyclodextrin grafted ammonium chitosan

Andrea Cesari[1, Φ], Alessandra Recchimurzo[1 Φ], Angela Fabiano[2], Federica Balzano[1, *], Nicolò Rossi[2], Chiara Migone[2], Gloria Uccello-Barretta[1], Ylenia Zambito[2] and Anna Maria Piras[2,*].

[1]Department of Chemistry and Industrial Chemistry, University of Pisa, via G. Moruzzi 13, 56126 Pisa, Italy; andrea.cesari@outlook.com (A.C.), alessandra.recchimurzo@phd.unipi.it (A.R.), federica.balzano@unipi.it (F.B.), gloria.uccello.barretta@unipi.it (G.U.B.)
[2]Department of Pharmacy, University of Pisa, via Bonanno Pisano 6, 56126 Pisa, Italy; angela.fabiano@unipi.it (A.F.), rossiniko94@hotmail.it (N.R), chiaramigone@gmail.com, ylenia.zambito@unipi.it (Y.Z), anna.piras@unipi.it (A.M.P).
* Correspondence: federica.balzano@unipi.it (F.B.); anna.piras@unipi.it (A.M.P.)

Φ The first two Authors equally contributed

Complexes stoichiometry (Job's Plot)

Table S1. Scheme of Job's Plot dilution series.

	DAL μM	MCD[a] μM
1	0	275.0
2	27.5	247.5
3	55.0	220.0
4	82.5	192.5
5	110.0	165.0
6	137.5	137.5
7	165.0	110.0
8	192.5	82.5
9	247.5	27.5
10	275.0	0

[a] MCD molarity considered for MCD, N^+-rCh-MCD and N^+-rCh /MCD solution series.

Evaluation of complex association constant (Benesi-Hildebrand method)

Table S2. Scheme of dilution series of DAL/MCD for the Benesi Hildebrand Plot – K_a determination by UV-VIS spectroscopy.

	Volume (mL)			Final Molarity (μM)	
Sample	DAL [0.4 mg/mL]	MCD [0.8 mg/mL]	H_2O	DAL	MCD
1	0.500	0.500	0	275	336
2	0.500	0.400	0.100	275	269
3	0.500	0.330	0.170	275	222
4	0.500	0.260	0.240	275	175
5	0.500	0.190	0.310	275	128

6	0.500	0.120	0.380	275	81
7	0.500	0.100	0.400	275	67
8	0.500	0	0.500	275	0

Table S3. Scheme of dilution series of DAL/MCD for the Benesi Hildebrand Plot – K_a determination by fluorescence spectroscopy.

	Volume (mL)			Final Molarity (µM)	
**Sample**	**DAL [0.1 mg/mL]**	**MCD [5 mg/mL]**	**H_2O**	**DAL**	**MCD**
1	0.500	0.500	0	70	2099
2	0.500	0.450	0.050	70	1889
3	0.500	0.400	0.100	70	1679
4	0.500	0.350	0.150	70	1469
5	0.500	0.300	0.200	70	1259
6	0.500	0.200	0.300	70	840
7	0.500	0.100	0.400	70	420
8	0.500	0.050	0.450	70	210
9	0.500	0	0.500	70	0

Table S4. Scheme of dilution series of DAL/N^+-Ch-MCD for the Benesi Hildebrand Plot – K_a determination by UV-VIS spectroscopy.

	Volume (mL)			Final Molarity (µM)	
**Sample**	**DAL [0.4 mg/mL]**	**N^+-Ch-MCD [grafted MCD 0.75 mg/mL]**	**H_2O**	**DAL**	**N^+-Ch-MCD**
1	0.500	0.500	0	275	311
2	0.500	0.350	0.150	275	218
3	0.500	0.260	0.240	275	162
4	0.500	0.190	0.310	275	118
5	0.500	0.120	0.380	275	75
6	0.500	0.100	0.400	275	62
7	0.500	0.050	0.450	275	31
8	0.500	0.020	0.480	275	12
9	0.500	0	0.500	275	0

Table S5. Scheme of dilution series of DAL/N^+-Ch-MCD for the Benesi Hildebrand Plot – K_a determination by fluorescence spectroscopy.

	Volume (mL)			Final Molarity (µM)	
**Sample**	**DAL [0.1 mg/mL]**	**N^+-Ch-MCD [grafted MCD 0.8 mg/mL]**	**H_2O**	**DAL**	**N^+-Ch-MCD**
1	0.20	0.20	0	70	332
2	0.20	0.18	0.02	70	300
3	0.20	0.16	0.04	70	266
4	0.20	0.14	0.06	70	233
5	0.20	0.12	0.08	70	200
6	0.20	0.10	0.10	70	166
7	0.20	0.08	0.12	70	133

8	0.20	0.05	0.15	70	83
9	0.20	0	0.20	70	0

NMR characterization of precursor N⁺-rCh and 2MCD

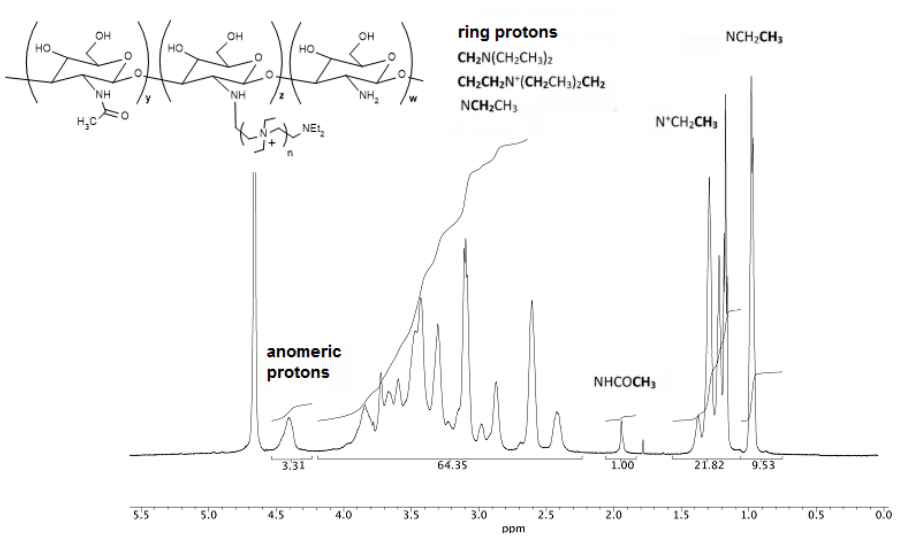

Figure S1. ^{1}H NMR spectrum (600 MHz, D_2O, 25 °C, 1.4 mg/mL) of ammonium-chitosan (N^+-rCh).

The degree of methylation of the cyclodextrin (DS=0.5), as declared by the supplier, was confirmed by proton NMR analysis of pure MCD (Fig. S2), by comparing the integrated areas of the anomeric protons H_1 and $H_{1'}$, respectively belonging to methylated and non-methylated units, which were assigned by exploiting scalar and dipolar correlations (COSY, TOCSY and ROESY 2D maps).

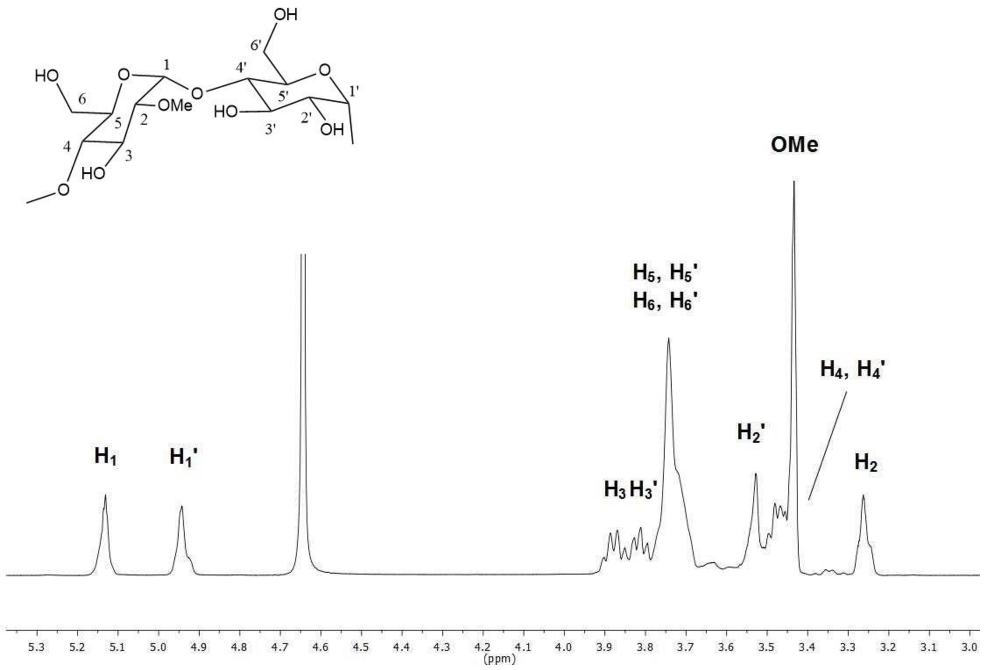

Figure S2. ^{1}H NMR spectrum (600 MHz, D_2O, 25 °C, 9.8 mM) of 2-methyl-β-cyclodextrin (2MCD).

Analysis protocol of N⁺-rCh-MCD

An average concentration of grafted cyclodextrin of 0.68 mM was determined in the NMR spectrum of the polymer (N^+-rCh-MCD) using an external standard of MCD with definite concentration (1.2 mM). Therefore, taking into account the average molecular weight of MCD (1191

g/mol), it was possible to calculate the average amount of cyclodextrin in the conjugated polymer (w^{gMCD}/w=22%). Since HDMI spacer covalently attached to ammonium-chitosan may not be fully capped with cyclodextrin and/or participate to inter- or intramolecular N^+-rCh chain-to-chain bonds, integrated areas of ^1H signals of N^+-rCh, MCD, and N^+-rCh-MCD were compared. Considering the ratio between the integrated areas of the anomeric and ring signals in the MCD spectrum and considering the ratio between the integrated area of the methyl signals and the remaining intermediate region (2.1-4.2 ppm) in N^+-rCh spectrum, the contribution deriving from the MCD and the precursor polymer were subtracted to the intermediate spectral region (2.0-4.5 ppm). In this way the integral contribution of two methylene groups of the spacer (10.72) and, therefore, the proton unit of the spacer were calculated (10.72, $^1H_{spacer}$=2.68). The ratio (5.8) between the proton units of the spacer and the cyclodextrin allows to determine the mass content of the spacer (18% w/w). Spacer/MCD ratio indicates that many spacer chains are bonded to the polymer, but not necessarily concatenated with the cyclodextrin.

NMR characterization of dalargin

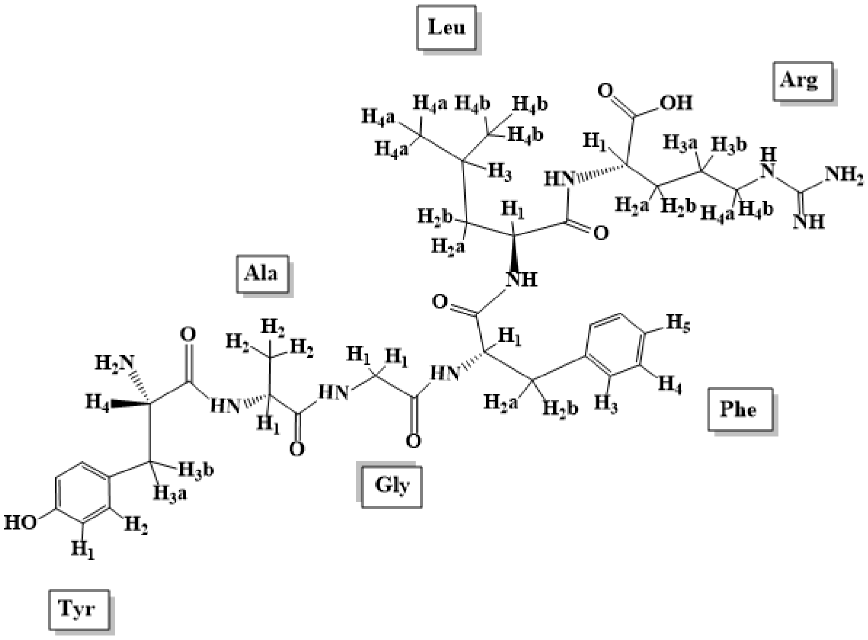

Figure S3. Structure of dalargin with numerated protons.

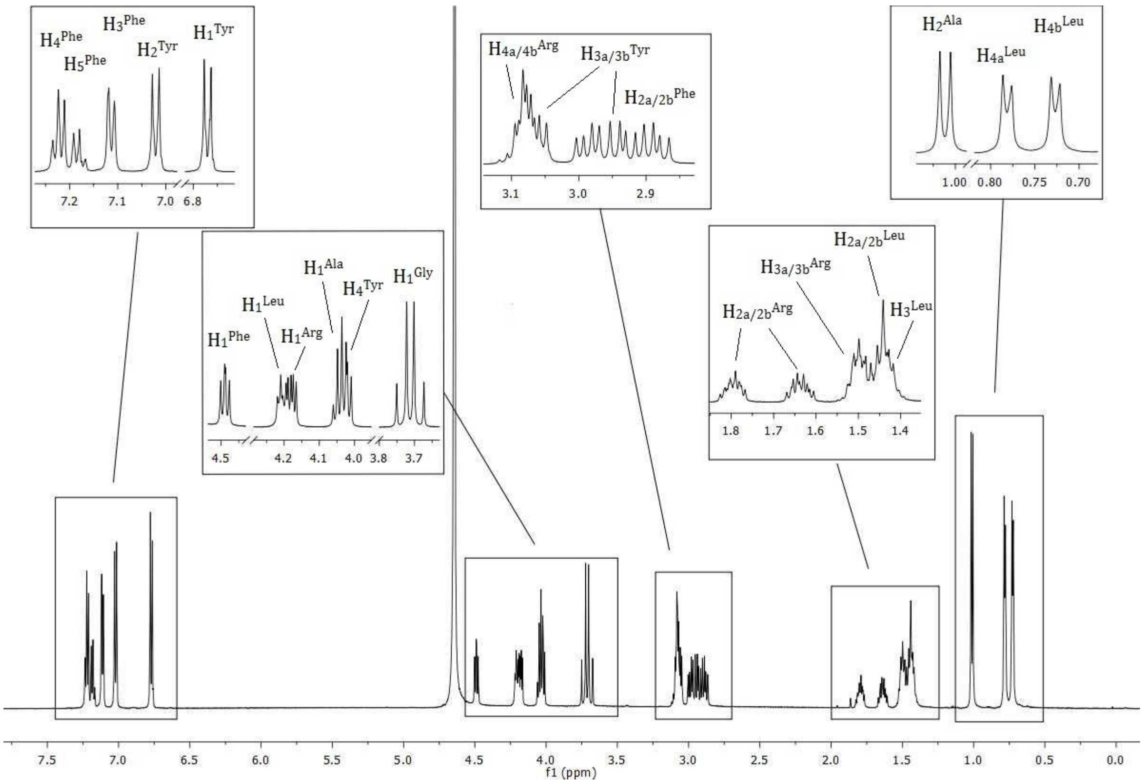

Figure S4. ^{1}H NMR spectrum (600 MHz, D_2O, 25 °C, 9.8 mM) of dalargin.

Table S6. ^{1}H NMR (600 MHz, D_2O, 25 °C, 9.8 mM) chemical shift of dalargin.

proton	δ (ppm)	proton	δ (ppm)
H_1^{Tyr}	6.77	H_1^{Leu}	4.21
H_2^{Tyr}	7.02	H_{2a}^{Leu} / H_{2b}^{Leu}	1.44
H_{3a}^{Tyr} / H_{3b}^{Tyr}	3.07/2.92	H_3^{Leu}	1.44
H_4^{Tyr}	4.03	H_{4a}^{Leu} / H_{4b}^{Leu}	0.78/0.72
H_1^{Ala}	4.04	H_1^{Arg}	4.17
H_2^{Ala}	1.00	H_{2a}^{Arg} / H_{2b}^{Arg}	1.79/1.63
H_1^{Gly}	3.71	H_{3a}^{Arg} / H_{3b}^{Arg}	1.50
H_1^{Phe}	4.49	H_{4a}^{Arg} / H_{4b}^{Arg}	3.09
H_{2a}^{Phe} / H_{2b}^{Phe}	2.99/2.90		
H_3^{Phe}	7.11		
H_4^{Phe}	7.22		
H_5^{Phe}	7.18		

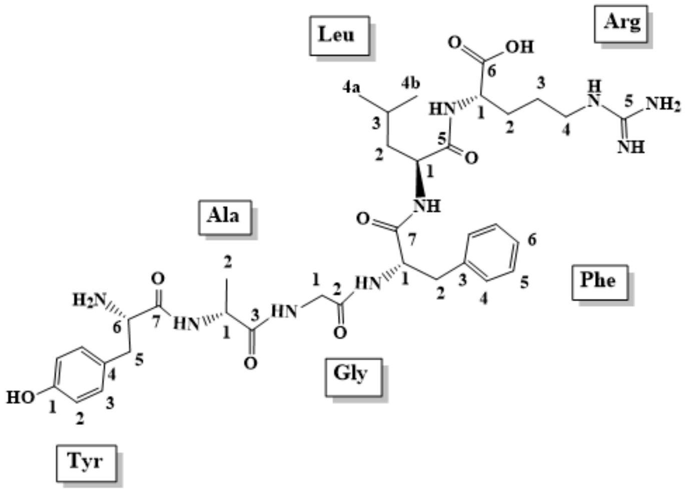

Figure S5. Structure of dalargin with numerated carbons.

Table S7. ^{13}C NMR (600 MHz, D_2O, 25 °C, 9.8 mM) chemical shift of dalargin.

Carbon	δ (ppm)	Carbon	δ (ppm)
C_1^{Tyr}	155.10	C_1^{Leu}	52.22
C_2^{Tyr}	115.77	C_2^{Leu}	39.69
C_3^{Tyr}	130.73	C_3^{Leu}	24.07
C_4^{Tyr}	125.50	C_{4a}^{Leu}	20.84
C_5^{Tyr}	35.98	C_{4b}^{Leu}	21.92
C_6^{Tyr}	54.49	C_5^{Leu}	173.77
C_7^{Tyr}	169.07	C_1^{Arg}	52.58
C_1^{Ala}	49.63	C_2^{Arg}	27.78
C_2^{Ala}	16.15	C_3^{Arg}	24.36
C_3^{Ala}	174.80	C_4^{Arg}	40.41
C_1^{Gly}	42.10	C_5^{Arg}	156.64
C_2^{Gly}	170.78	C_6^{Arg}	175.45
C_1^{Phe}	54.70		
C_2^{Phe}	36.86		
C_3^{Phe}	136.04		
C_4^{Phe}	129.09		
C_5^{Phe}	128.66		
C_6^{Phe}	127.11		
C_7^{Phe}	172.68		

Table S8. Molar fraction (χ) determined by Job's Plot. χ = [DAL]/([DAL] + [MCD]).

	Incubation time (min)	Molar fraction (χ)	R^2
DAL/MCD	0	0.500	0.999
	1h	0.490	0.995
	24h	0.480	0.995
DAL/N^+-Ch / MCD	0	0.500	0.997
	1h	0.520	0.969
	24h	0.470	1
DAL/N^+-Ch-MCD	0	0.500	0.987

1h	0.490	0.985
24h	0.560	0.994

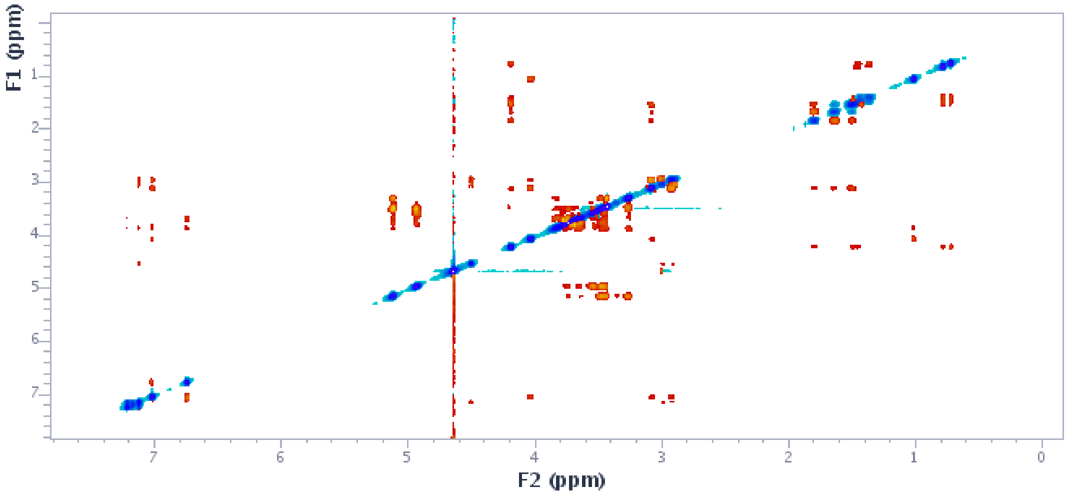

Figure S6: ROESY map (600 MHz, D_2O, 25 °C, 9.8 mM) of DAL/MCD mixture (1:1).

In-vitro Caco-2 Monolayer

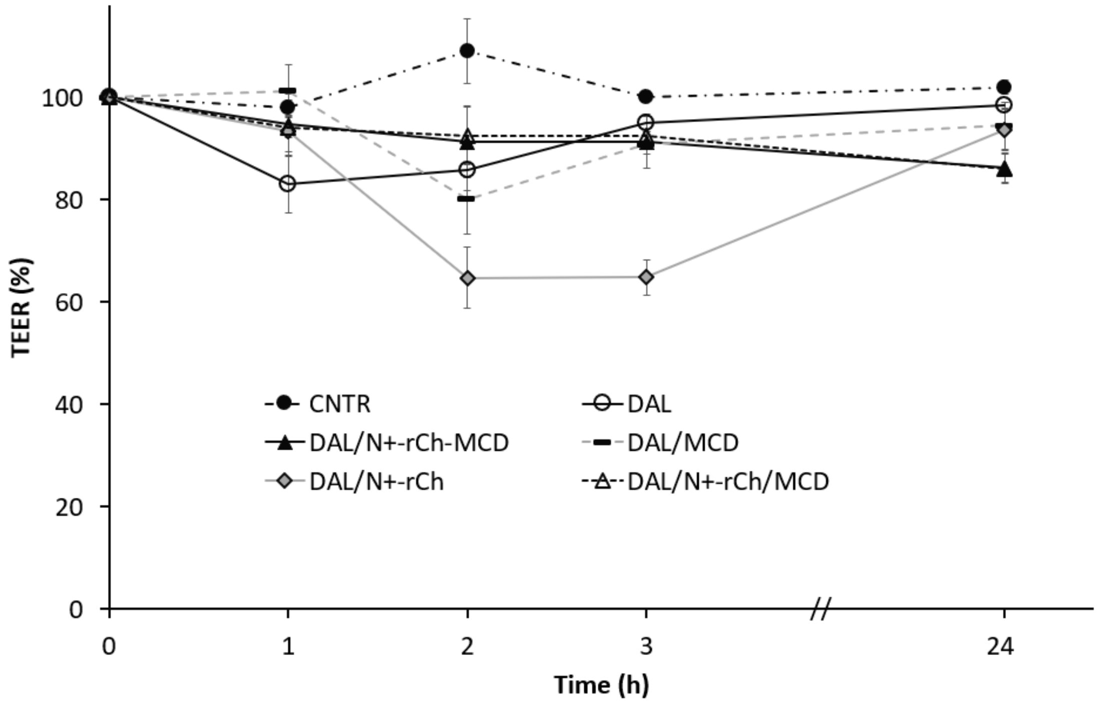

Figure S7: The transepithelial electrical resistance (TEER, Ωcm^2) across Caco-2 monolayer.
